# Bio immune(G)en medicine in allergic diseases especially asthma

**DOI:** 10.1186/1939-4551-8-S1-A58

**Published:** 2015-04-08

**Authors:** Gilbert Glady

**Affiliations:** 1 EBMA, France

## Background

Bio Immune(G)en Medicine is a diagnostic and therapeutic method representing a new immunomodulating way of approaching the asthmatic disease.

## Methods

To reach diagnosis this method uses a wide range of biological parameters providing a great deal of significant information in the context of allergy such as lymphocyte typing and protein profiles or the Th1/Th2 differentiation, as well as many bacterial and viral serologies, to substantiate the biological diagnosis and guide the therapeutic result.

Within this therapy, numerous immune-competent signaling molecules are used, especially microRNAs representing actually one of the best known epigenetic process, which are prepared according to the homeopathic dilution-dynamisation mode to ensure total safety in terms of any adverse side-effects.

## Results

This allows developing of a biomimetic and holistic immune therapy involving the neutralization of certain microbial agents, of which regulatory, medium-to-long-term effects are proving to be extremely beneficial to patients in a large number of allergic diseases.

## Conclusions

The description of some clinical cases of asthma will help to highlight the way in which this innovative method links with effectiveness the findings of basic research to human clinic and therapeutics.

